# Epstein-Barr virus-associated lymphoma of the orbit: Case report and review of the literature

**DOI:** 10.1016/j.ajoc.2022.101628

**Published:** 2022-06-20

**Authors:** Michael Chang, Natalie Hobeika, Bradley A. Thuro

**Affiliations:** a Department of Ophthalmology and Visual Sciences at West Virginia University, Morgantown, WV, USA; b West Virginia University School of Medicine, Morgantown, WV, USA

**Keywords:** Orbital tumor, Diffuse large B-Cell lymphoma, Epstein-barr virus

## Abstract

**Purpose:**

Herein, the authors describe an unusual presentation of EBV-associated diffuse large B-cell lymphoma in the bilateral orbits of an immunocompetent woman.

**Observations:**

An elderly, immunocompetent woman presented with an orbital mass which was biopsied and proven to be EBV-associated lymphoma. The authors then performed a literature search and review which suggested this presentation to be rare as it has been infrequently reported in the ophthalmic literature.

**Conclusions and importance:**

More research is needed to fully ascertain the importance of EBV in ocular adnexal lymphoma. The authors hope this case report adds to the body of literature a different presentation of EBV-associated ocular adnexal lymphoma.

Epstein- Barr virus (EBV) is a ubiquitous virus, infecting over 90% of humans worldwide.^1^^,^^2^ Classically associated with Burkitt and Hodgkin lymphomas, EBV has also been linked to other lymphoid malignancies, especially in individuals with congenital or acquired immunodeficiencies resulting in impaired T-cell immunity.^1^^,^^3^ However, EBV- associated tumorigenesis has also been implicated in immunocompetent individuals. EBV-positive diffuse large B-cell lymphoma (DLBCL) is an aggressive type of non-Hodgkin lymphoma (NHL) that is rarely described in the ocular adnexal region. Herein, we report a case of EBV-positive orbital DLBCL in an immunocompetent patient. Patient data were collected in compliance with the Health Insurance Portability and Accountability Act (HIPAA) and was done in a manner consistent with the tenets as outlined in the Declaration of Helsinki. Formal Institutional Review Board (IRB) determined exemption from requiring informed consent.

## 1 Case report

**Fig. 1:**
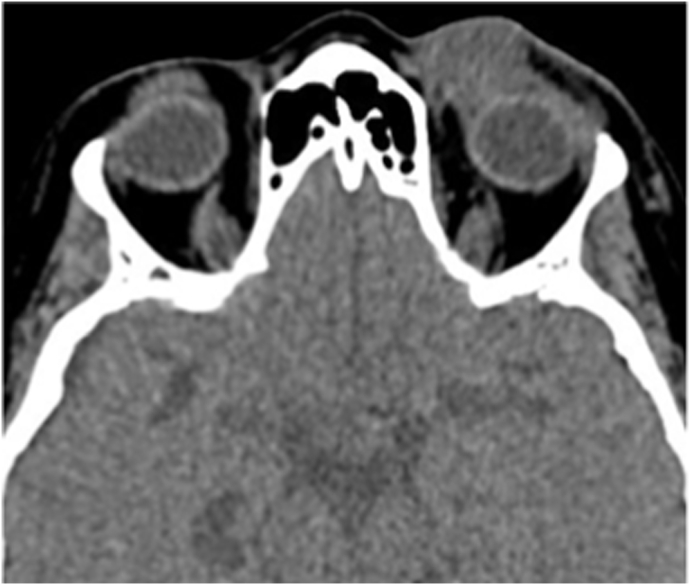
Initial presentation of an elderly Caucasian female with EBV-positive DLBCL. CT scan showing a left orbital lesion corresponding to the left upper lid lesion.

A 77-year-old Caucasian female presented to the emergency department with a 3-week history of worsening left upper eyelid edema and erythema. Symptoms continued to progress despite a 4-day course of trimethoprim/sulfamethoxazole for suspected cellulitis. She denied a personal history of eye trauma or malignancy. Examination revealed an erythematous, non-fluctuant and non-mobile, painless lesion along the nasal aspect of the left upper eyelid. Computerized tomography (CT) of the orbits without contrast revealed a homogenous left upper lid soft tissue lesion (shown in Fig. 1). She underwent an anterior orbitotomy with incisional biopsy of the left orbital mass.

**Fig. 2:**
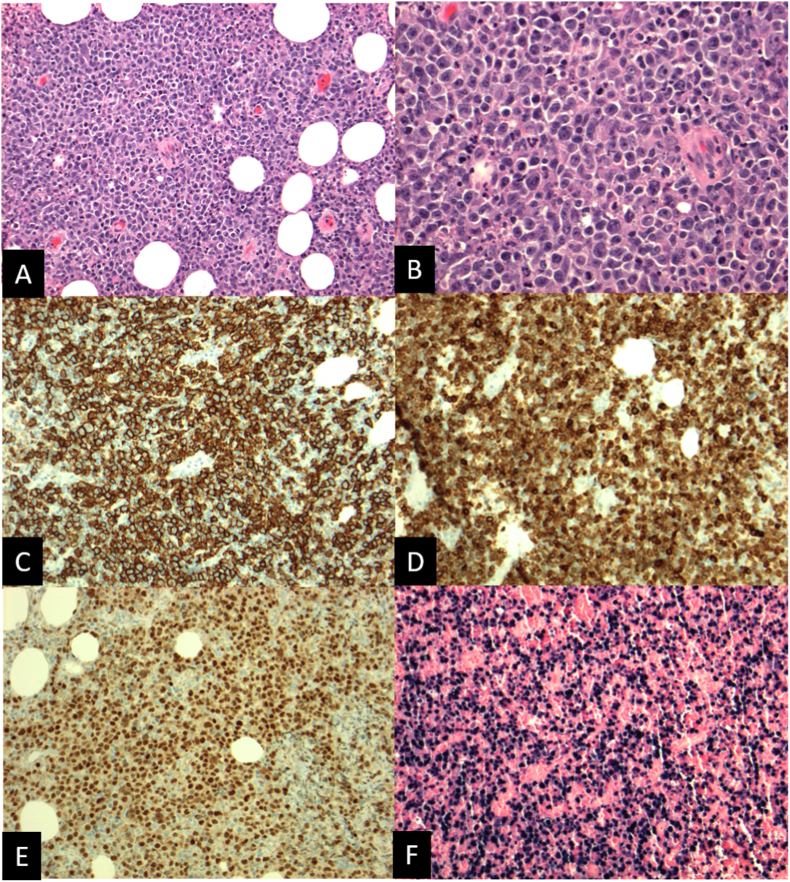
Histologic examination of biopsied specimen following orbitotomy. Intermediate-power (20x) magnification with hematoxylin-eosin staining shows diffuse lymphoid infiltrate with geographic areas of necrosis (A). High-power (40x) magnification shows large, pleomorphic lymphocytic cells (B). Immunohistochemistry confirmed expression of B-cell markers including CD20 (C), CD79a (D), and PAX5 (E). In situ hybridization also showed positivity of the EBER, confirming diagnosis if EBV-associated disease (F).

Microscopically, the biopsied specimen showed sheets of large atypical lymphoid cells with geographic necrosis (shown in Fig. 2A and B). By immunohistochemistry, the malignant cells expressed CD20 (shown in Fig. 2C), CD79a (shown in Fig. 2D), and PAX 5 (shown in Fig. 2E). Cells also showed positivity with Epstein-Barr encoding region (EBER) by in situ hybridization. (shown in Fig. 2F). Fluorescent in situ hybridization (FISH) was negative for MYC or BCL6 rearrangements and IGH/BCL2 fusion. These findings were suggestive of a diagnosis of EBV-positive DLBCL of the orbit.

Staging performed with positron emission tomography (PET) showed bilateral orbital involvement which was not previously appreciated on the non-contrast enhanced CT orbits; systemic involvement was detected in the left superior trapezius muscle and bilateral upper extremities— demonstrating stage IV disease by the Lugano Classification/modified Ann Arbor Classification system and T4N3M1a classification by the American Joint Committee on Cancer (AJCC) staging system. After 8 months of follow-up, she has completed four of six cycles of rituximab, cyclophosphamide, doxorubicin, vincristine, and prednisone (R–CHOP) therapy. Repeat PET scan after four treatments showed complete response of her disease.

A literature search was then performed using PubMed and MEDLINE databases for any articles containing the terms “ocular adnexal lymphoma,” “orbital lymphoma,” and “Epstein-Barr virus.” The references of each queried article were then further evaluated.

## 2 Discussion

Ocular adnexal lymphomas (OAL) constitute less than 1% of all NHLs.^4^ However, they are the most common primary orbital malignancy in the United States, accounting for 55% of all orbital malignancies.^5^ DLBCL comprises 5–15% of OALs, with most cases affecting the orbit.^6^ EBV-associated DLBCL has a reported prevalence in Eastern Asian of 8–11% of DLBCL cases, compared to less than 5% of DLBC cases in Western countries.^2^^,^^7^ In a study by Ahmed et al., of 396 patients with ophthalmic DLBCL, approximately 60% of patients had orbital involvement with only 2.1% of those cases presenting with bilateral orbital disease.^8^ While this study was not specific to EBV-positive DLBCL, it highlights the rarity of bilateral orbital DLBC. Thus, our case is unique not only in its presentation in a Caucasian patient, but also in its bilateral orbital involvement.

OALs often present both diagnostic and treatment challenges. Due to often non-specific clinical features, as well as late presentation of symptoms, the diagnosis of OALs is often delayed. Compared to EBV-negative DLBCL, EBV-associated lymphoproliferative disorders (LPD), as a group, have been shown to present with more aggressive clinical features. In a study by Oyama et al., EBV-positive LPDs present with significantly higher International Prognostic Index (IPI) compared to EBV-negative DLBCL, with 54% of EBV-positive cases in the high or high intermediate risk group.^9^ Furthermore, even when accounting for age, the overall survival of EBV-associated LPD was inferior to that of EBV-negative DLBCL. While EBV-positive LPDs have been described in Asian individuals, there are very few studies focused on Western populations. A case series of 5 patients with EBV-positive B-cell lymphomas in the United States showed similarly poor clinical outcomes with 3 deaths within 22 days to 20 months of diagnosis.^10^ The remaining 2 patients initially showed response to R–CHOP therapy, but subsequently developed recurrence. Our patient appears to have shown good initial response while undergoing chemotherapy— however, her long-term outcome is still to be determined.

We present a case of bilateral orbital EBV-positive DLBCL in an elderly, otherwise immunocompetent, Caucasian female who showed response to R–CHOP chemotherapy. Due to its rarity, larger population studies to further characterize clinical features and mortality of EBV-positive DLBCL is difficult. Further investigation of EBV-positive DLBCL, particularly in Western countries, is needed to further classify population-based differences in clinical features and prognosis of the disease.

## Statement of ethics

This study protocol was reviewed and approved by the WVU IRB, approval number 2107355074. The IRB determined the study to be exempt from further ethical oversight. The IRB also determined that written informed consent was not required and was thus waived.

## Funding sources

No funding sources were required or used in this study.

## Author contributions

Michael Chang: Initial data acquisition and review of relevant literature, along with significant contribution to the writing and editing of the manuscript.

Natalie Hobeika: Initial data acquisition and review of relevant literature, along with significant contribution to the writing and editing of the manuscript.

Bradley Thuro: Review of the relevant literature, along with significant contribution to writing and editing of the manuscript, with final approval.

## Data availability statement

All data generated or analyzed during this study are included in this article and its supplementary reference list. Further enquiries can be directed to the corresponding author.

## Patient consent

Written consent to publish this case has not been obtained. This report does not contain any personal identifying information.

## Declaration of competing interest

The authors have no conflicts of interest to declare.
